# Melatonin Ameliorates Ovarian Hyperstimulation Syndrome (OHSS) through SESN2 Regulated Antiapoptosis

**DOI:** 10.1155/2023/1121227

**Published:** 2023-10-28

**Authors:** Min Zheng, Mei Liu, Cong Zhang

**Affiliations:** ^1^Center for Reproductive Medicine, Ren Ji Hospital, School of Medicine, Shanghai Jiao Tong University, Shanghai, China; ^2^Shanghai Key Laboratory for Assisted Reproduction and Reproductive Genetics, Shanghai, China; ^3^Department of Obstetrics, Affiliated Hospital of Shandong University of Traditional Chinese Medicine, Ji'nan, Shandong, China; ^4^Shandong Provincial Key Laboratory of Animal Resistance Biology, College of Life Sciences, Shandong Normal University, Ji'nan, Shandong, China

## Abstract

**Background:**

Ovarian hyperstimulation syndrome (OHSS) is one of the most severe complications after ovarian stimulation during assisted reproductive technology (ART). However, its pathogenesis still remains unclear. Melatonin is an important antioxidant factor in female reproduction and Sestrin-2 (SESN2) is reported to be involved in cellular response to different stress conditions. Whether or not melatonin and SESN2 are involved in OHSS is still a question to us clinicians.

**Methods and Results:**

We collected the granulosa cells of OHSS patients and focused on the role of SESN2 in OHSS. We also studied the role and mechanism of melatonin plays in OHSS patients. We found that the expression of SESN2 was increased in the granulosa cells of OHSS patients (*n* = 24) than those in controls (*n* = 15). Incubation with angiotensin II (1 *μ*M, 2 *μ*M) in HUVECs and H2O2 (0.1 mM, 0.2 mM) in KGNs increased the generation of ROS concurrent with the increased expression of SESN2, while melatonin treatment partly restored SESN2 levels. The mechanism study demonstrated that SESN2 was deeply involved in the regulation of AMPK and mTOR, whereas melatonin partially restored angiotensin II or H2O2 induced the activation of AMPK phosphorylation and the inhibition of mTOR, 4EBP1 and S6K1 phosphorylation, all of which could trigger cell apoptosis.

**Conclusions:**

These findings indicated that melatonin attenuated ROS-induced apoptosis through SESN2-AMPK-mTOR in OHSS. Thus, melatonin is likely to be a potential and important therapeutic agent for treating and preventing OHSS.

## 1. Introduction

Infertility is defined as the inability to be pregnant within one year of unprotected intercourse and its incidence has reached to 10–15% in recent years [1]. It has become not only a medical concern but also a social issue with increasing prevalence in both developed and developing countries. In vitro fertilization (IVF) is wildly accepted over the past 35 years as an effective treatment for infertility during which controlled ovarian stimulation (COH) is almost always employed to retrieve more oocytes. Although COH might improve IVF outcome, it also increases the risk of an iatrogenic complication: ovarian hyperstimulation syndrome (OHSS). OHSS is not uncommon. Studies reported that the presence of its moderate and severe form was up to 10% of all IVF cycles [2]. The true incidence of OHSS is probably much underestimated since the symptoms of mild OHSS are easy to be ignored [3]. The symptoms of OHSS in its mild form can be untypical such as nausea and vomiting; however, moderate and severe OHSS may result in oliguria, hydrothorax, ascites, hepatorenal failure, acute respiratory distress syndrome, hemorrhage from ovarian rupture, thromboembolism, and ultimately, even death [4]. Although OHSS increases the physical, psychological, and economic burden of the patients and their families, its pathogenesis is not completely understood and no specific therapy is available for this syndrome [5, 6]. Therefore, prevention of OHSS becomes a crucial issue since its treatment is largely tamping down symptoms, rather than addressing causes. So far, several strategies including cycle cancellation, coasting, in vitro oocyte maturation have been used in practice to prevent OHSS. Ismet H et al. have shown that oxytocin as well as cabergoline alleviates OHSS in OHSS rat models [7]. More studies should be focus on the prevention of OHSS.

The precise cause of OHSS remains currently the subject of controversy. Nevertheless, high estradiol levels in the presence of human chorionic gonadotropin (hCG) increase the vascular endothelium permeability, leading to a massive shift of intravascular fluid into the third space. There are also evidences that during the pathogenesis of this iatrogenic complication, large amounts of angiotensin II, vascular endothelial growth factor (VEGF), interleukins (ILs), nitric oxide (NO), tumor necrosis factor-a (TNF-a), and other molecules are excessively produced, causing the overproduction of reactive oxygen species (ROS) which results in oxidant-antioxidant imbalance [8]. The vascular endothelium is then deteriorated by these imbalanced free radicals that cannot be antagonized by free radical scavengers; consequently, high vascular permeability occurs and finally results in the aggravation of OHSS [9].

Melatonin is mainly secreted by pineal glands in human beings and is regulated by circadian rhythms. However, higher levels of melatonin are found in human follicular fluid than in plasma because melatonin is not only derived from the general circulation but also synthesized in the ovary (mainly by the granulosa cells) [10]. Melatonin has a significant impact on female reproduction. It is considered essential for folliculogenesis, steroid production. There are also evidences that melatonin takes part in the control of pubertal onset, ovulation, sexual maturation and pregnancy protection [11]. Melatonin as well as its metabolites has been proved to be a powerful radical scavenger. It reduces ROS levels in the ovary through receptor dependent and independent pathways [12, 13]. Recently, more and more attention has been paid to the importance of melatonin in female reproduction [14].

Sestrins are highly conserved and stress-inducible metabolic regulators which are ubiquitously expressed at different levels in all adult tissues [15, 16]. The physiological functions of sestrins have not been fully elucidated yet. The critical roles of sestrins in mammalian metabolism have been revealed by the deletion of these proteins which is incompatible with the survival of mice [17]. Previous studies have also suggested that sestrins have a close relationship with age and oxidative stress associated diseases such as Alzheimer's disease, Parkinson's disease, and diabetes and have a favorable profile as potential therapeutic targets for these diseases [18, 19]. Sestrin-2 (SESN2) belongs to the sestrins family and functions as a suppressor of ROS accumulation as well as a neuroprotector [20]. The overexpression of SESN2 reduces ROS levels whereas SESN2 knockdown in cultured cells or mice increases ROS content [21, 22]. Moreover, any condition that leads to ROS accumulation induces SESN2 expression [19]. Therefore, the increased ROS levels in OHSS may increase the expression of SESN2. Most previous studies on SESN2 have been focused on the nervous system and cardiovascular system; these studies have shown that SESN2 played an important role in preventing ROS damage, repairing mitochondria deterioration and maintaining the stability of inner environment [23, 24]. However, there are very few studies on the expression and the function of SESN2 in human reproduction. Since OHSS is closely associated with the excessive production of ROS and melatonin is supposed to be a powerful radical scavenger, the objective of this study was to investigate whether SESN2 are induced in OHSS and whether melatonin can alleviate oxidative stress in OHSS as well as the potential role of SESN2 in OHSS.

## 2. Materials and Methods

### 2.1. Clinical Sample Collection

The diagnosis of OHSS is based on the guideline of OHSS by Practice Committee of the American Society for Reproductive in 2016 [25]. Patients present with abdominal distention, nausea, vomiting and relevant results of laboratory investigations or ultrasound scan are diagnosed as OHSS during our ART procedures after being excluded of other diseases. The severe symptoms of OHSS include oliguria, anuria, ascites, hydrothorax and thromboembolism, acute respiratory distress syndrome. Clinical parameters including age, BMI, AMH, basal FSH, basal *E*_2_, *E*_2_ on hCG administration day, number of oocytes retrieved were collected. Patients received hCG when the diameter of their follicles was >18 mm. Oocytes were collected 36 h after hCG injection by transvaginal ultrasound-guided puncture and aspiration of the follicles with a diameter of 18 to 20 mm. The granulosa cells from patients on oocyte retrieval day were collected and purified with Ficoll-Pague™ PLUS (GE-HealthCare Bio-Science, Sweden). Our research was approved by the Reproductive Ethics Committee of Ren Ji Hospital (No. 2015030308). The informed consent was obtained from each patient before oocyte retrieval.

### 2.2. Cell Culture

Human umbilical vein endothelial cells (HUVECs) and human granulosa cell line (KGNs) were kindly provided by Shandong University. HUVECs represent endothelial cells and KGNs are used to represent ovarian granulosa cells in previous studies [26].

### 2.3. The Evaluation of Intracellular ROS of Cultured Cells

Intracellular ROS levels were measured using fluoroprobe CM-H_2_DCFDA (Sigma-Aldrich, Saint Louis, Missouri, USA). Cells cultured in six-well plates were incubated in DMED/F12 with 10 mM CM-H_2_DCFDA for 20 min at 37°C in a dark, humidified chamber. CM-H_2_DCFDA fluorescence was measured using a fluorescence microscope (Zeiss, Germany) with a digital imaging system at an excitation wavelength ranging from 430 to 480 nm and the intensity of the fluorescence were analyzed using IMAGE J 1.34 s (National Institutes of Health, USA).

### 2.4. Statistical Analysis RNA Extraction and Real-Time PCR

The expression of target genes was detected by real-time polymerase chain reaction and the results were analyzed by ^ΔΔ^Ct methods [27]. *ß*-actin was chosen as an internal control. The sequences of the primers used for amplification are:

  SESN2 5′-TCTTACCTGGTAGGCTCCCAC-3′
  5′-AGCAACTTGTTGATCTCGCTG-3′.
  ATCB 5′-CTCCATCCTGGCCTCGCTGT-3′
  5′-GCTGTCACCTTCACCGTTCC-3.

### 2.5. Western Blot

30 *μ*g proteins were applied to an SDS gel for electrophoresis and were then transferred to polyvinylidene fluoride membranes. Nonspecific binding sites were blocked using 5% non-fat milk for 90 min at room temperature. After blocking, the membrane was incubated overnight at 4°C with the following primary antibodies: anti-SESN2 antibody (1 : 1000, Proteintech Group Inc, Chicago, Illinois, USA), anti-p-S6K1Thr389 antibody (1 : 1000, Cell Signaling Technology, Danvers, Massachusetts, USA), anti-S6K1 antibody (1 : 1000, Cell Signaling Technology, Danvers, Massachusetts, USA), anti-BCL2 antibody (1 : 1000, Proteintech Group Inc, Chicago, Illinois, USA), anti-p-4EBP1^Thr37/46^ antibody (1 : 1000, Cell Signaling Technology, Danvers, Massachusetts, USA), anti-p-mTOR^Ser2448^ antibody (1 : 1000, Cell Signaling Technology, Danvers, Massachusetts, USA), anti-mTOR antibody (1 : 1000, Cell Signaling Technology, Danvers, Massachusetts, USA), anti-p-AMPK^Thr173^ antibody (1 : 1000, Cell Signaling Technology, Danvers, Massachusetts, USA), anti-AMPK antibody (1 : 1000, Cell Signaling Technology, Danvers, Massachusetts, USA), anti-p17 caspase 3 antibody (1 : 1000, Proteintech Group Inc., Chicago, Illinois, USA), anti-caspase 3 antibody (1 : 1000, Proteintech Group Inc., Chicago, Illinois, USA), and anti-GAPDH antibody (1 : 1000, Proteintech Group Inc., Chicago, Illinois, USA). The bands were then analyzed using Quantiscan software (Biosoft, UK) [28–30].

### 2.6. Statistical Analysis

All data were analyzed using SPSS 22.0 software (IBM, Armonk, New York, USA). The differences between two groups were analyzed using Student's t-test. The differences among three groups or above were analyzed using one-way ANOVA analysis followed by the Newman–Keuls multiple comparison test. The data are represented as mean ± standard deviation (SD) and all experiments were repeated at least three times. *P* < 0.05 was regarded as statistically significant.

## 3. Results

### 3.1. OHSS Patients Showed an Increased Expression of SESN2 in Granulosa Cells

**Figure 1:**
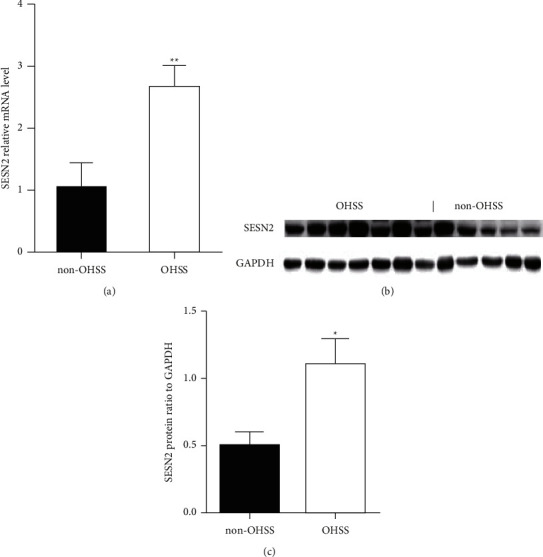
The expression of SESN2 in granulosa cells of OHSS patients and controls. (a) The expression of SESN2 mRNA in granulosa cells of OHSS (*n* = 24) patients and controls (*n* = 15) (*t* = −3.231, *P*=0.003); (b) the representative pictures of Western blotting of SESN2 and GAPDH in the granulosa cells of OHSS patients and controls; (c) the comparison of SESN2/GAPDH protein ratio in the granulosa cells of OHSS patients (*n* = 24) and controls (*n* = 15). ^*∗*^*P* < 0.05; ^*∗∗*^*P* < 0.01.

The clinical characteristics of the patients are presented in Table 1. The OHSS patients had younger age, lower BMI, lower base FSH levels than the women in non-OHSS group and higher AMH levels, higher *E*_2_ levels on hCG day; moreover, OHSS patients retrieved more oocytes than non-OHSS group (*P* < 0.05). Furthermore, the suppressor of ROS accumulation, SESN2, both its mRNA (OHSS *n* = 24, control *n* = 15) (*P*=0.003) and protein levels (OHSS *n* = 24, control *n* = 15) (*P*=0.038) were significantly increased in the granulosa cells of OHSS patients (Figures 1(a) and 1(b)).

### 3.2. SESN2 Levels Were Parallel to the ROS Generation in HUVECs and KGNs

**Figure 2:**
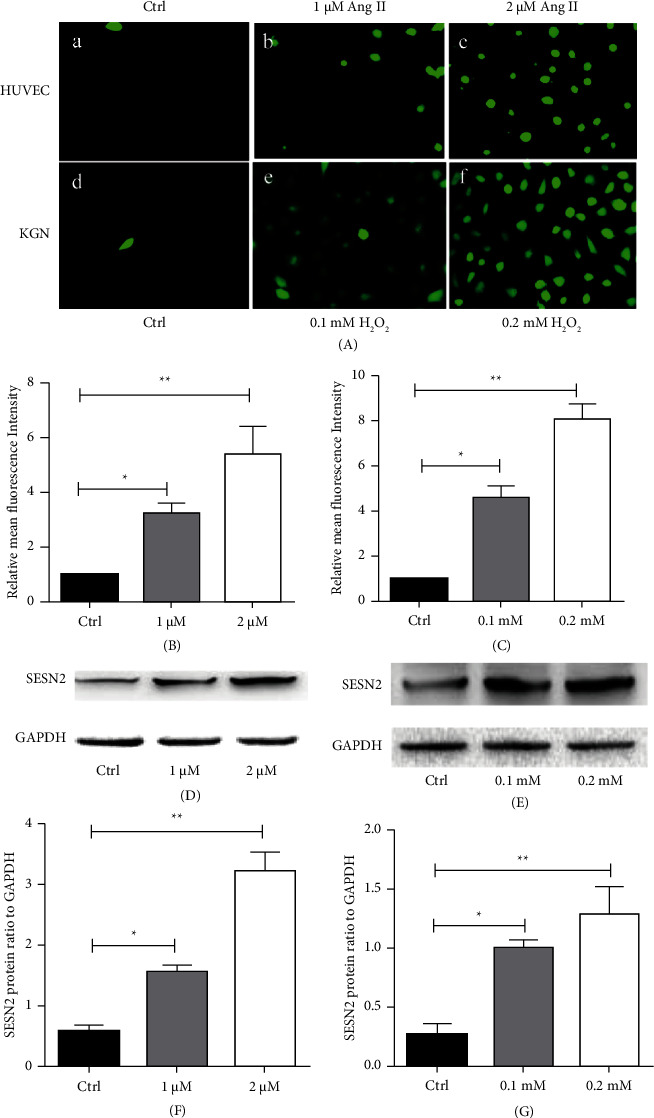
The oxidative stress and SESN2 levels in HUVECs and KGNs induced by different concentrations of angiotensin II or H_2_O_2_. (A) (a–c) the representative pictures of controls, 1 *μ*M, 2 *μ*M angiotensin II treated HUVECs dyed with DCFH-DA, respectively; (d–f) the representative pictures of controls, 0.1 mM, 0.2 mM H_2_O_2_ treated KGNs dyed with DCFH-DA, respectively; (B) the comparison of mean oxidative fluorescence intensity in HUVECs. The mean of the results in controls was assigned an arbitrary value of 1.0 and the results (mean ± SD) of other cells are expressed as the relative intensity; (C) the comparison of mean oxidative fluorescence intensity in KGNs. The mean of the results in controls was assigned an arbitrary value of 1.0 and the results (mean ± SD) of other cells are expressed as the relative intensity. Bar: 50 *μ*m; (D) the representative picture of Western blotting of SESN2 and GAPDH in controls, 1 *μ*M, 2 *μ*M angiotensin II treated HUVECs; (E) the representative picture of Western blotting of SESN2 and GAPDH in controls, 0.1 mM, 0.2 mM H_2_O_2_ treated KGNs; (F) the comparison of SESN2/GAPDH ratio after being incubated with angiotensin II for 24 h; (G) the comparison of SESN2/GAPDH ratio after incubation with H_2_O_2_ for 12 h. The results are expressed as mean ± SD. ^*∗*^*P* < 0.05; ^*∗∗*^*P* < 0.01; ^*∗∗∗*^*P* < 0.001. Ctrl: control.

Since OHSS is highly associated with the excessive production of ROS, which induces the deterioration of endothelial cells and the dilatation of vessels in whole body, we explored SESN2 and ROS levels in OHSS. We chose HUVECs as endothelial cells model and had them cultured with Angiotensin II to build OHSS oxidative stress model. Incubation with angiotensin II (1 *μ*M, 2 *μ*M) significantly increased the generation of ROS (1 *μ*M angiotensin II versus control: 3.53 ± 0.28 versus 1.00, *P*=0.034; 2 *μ*M angiotensin II versus control: 5.46 ± 0.98 versus 1.00, *P*=0.005) (Figures 2(A) and 2(B)). The SESN2 levels were also significantly increased (*P*=0.042) (Figures 2(D) and 2(F)). Therefore, the SESN2 levels were in parallel to the ROS generation in HUVECs.

To further confirm the results observed, we also used KGNs cells to mimic ovarian granulosa cells. The same phenomenon was observed in KGNs cells. The ROS generation and SESN2 levels in cultured KGNs cells were significantly increased after incubation with H_2_O_2_ (0.1 mM, 0.2 mM) (Figures 2(A) and 2(C), 2(E), 2(G)).

### 3.3. Melatonin Significantly Restored SESN2 Levels in Cultured HUVECs and KGNs

**Figure 3:**
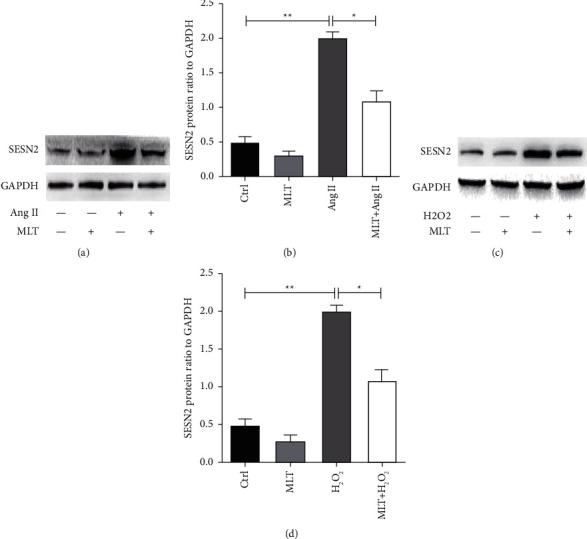
The effect of melatonin on SESN2 levels of HUVEC and KGNs incubated with angiotensin II or H_2_O_2_. (a) The representative pictures of Western blotting of SESN2 and GAPDH in controls, 10 *μ*Μ melatonin or 1 *μ*M angiotensin II treated cells before incubation with 1 *μ*M angiotensin II in HUVECs; (b) the comparison of SESN2/GAPDH ratio in controls, angiotensin II, melatonin treated HUVECs; (c) the representative pictures of Western blotting of SESN2 and GAPDH in controls, 1 mΜ melatonin, 0.1 mM H_2_O_2_, 1 mΜ melatonin treated cells before incubation with 0.1 mM H_2_O_2_ in KGNs; (d) the comparison of SESN2/GAPDH ratio in controls, melatonin, H_2_O_2_, treated KGNs. The results are expressed as mean ± SD. ^*∗*^*P* < 0.05; ^*∗∗*^*P* < 0.01; ^*∗∗∗*^*P* < 0.001. Ctrl: control; MLT: melatonin; Ang II: angiotensin II.

Since ROS and SESN2 levels are increased in OHSS model and melatonin is a powerful radical scavenger, we explored the role of melatonin in treating OHSS. The treatment with melatonin (10 *μ*M) for 24 h significantly decreased SESN2 level induced by angiotensin II (*P*=0.037) (Figures 3(a) and 3(b)), similarly, the treatment with melatonin (1 mM) for 12 h significantly decreased SESN2 level in KGNs cells induced by H_2_O_2_ (Figures 3(c) and 3(d)).

### 3.4. Melatonin Inhibited Apoptosis through SESN2 Regulated Signaling Pathway in HUVECs and KGNs

**Figure 4:**
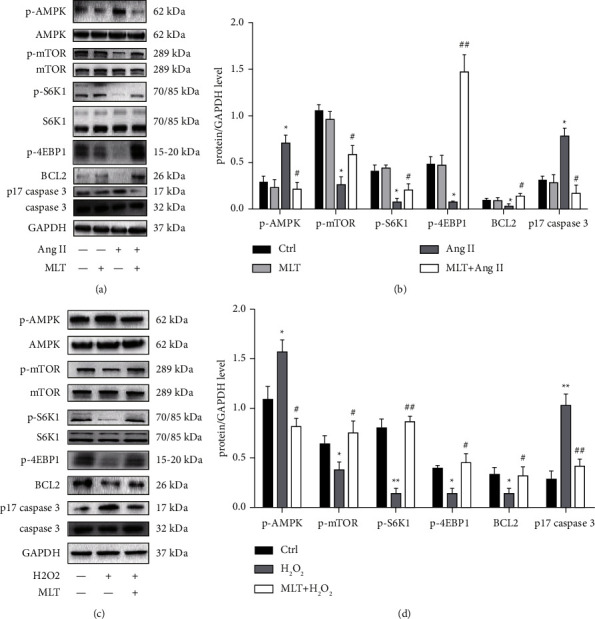
The effect of melatonin on apoptosis related proteins in HUVECs and KGNs incubated with angiotensin II or H_2_O_2_. (a) The representative pictures of Western blotting of p-AMPK, AMPK, p-mTOR, mTOR and its downstream factors (p-S6K1, S6K1, p-4EBP1) as well as apoptosis related molecules (p17 caspase 3, caspase 3, BCL2) and GAPDH in melatonin and angiotensin II treated HUVECs; (b) the comparison of protein/GAPDH ratio in HUVECs; (c) the representative pictures of Western blotting of p-AMPK, AMPK, p-mTOR, mTOR and its downstream factors (p-S6K1, S6K1, p-4EBP1) as well as apoptosis related molecules (p17 caspase 3, caspase 3, Bcl2) and GAPDH in melatonin and H_2_O_2_ treated KGNs; (d) the comparison of protein/GAPDH ratio in KGNs. The results are expressed as mean ± SD. ^*∗*^*P* < 0.05 vs. control group; ^*∗∗*^*P* < 0.01 vs. control group; ^#^*P* < 0.05 vs. angiotensin II group; ^##^*P* < 0.01 vs. angiotensin II group. Ctrl: control; MLT: melatonin; Ang II: angiotensin II.

Previous studies have demonstrated that melatonin plays an antioxidant role and regulates antiapoptotic pathway [31–34]. To better investigate the role and mechanism melatonin plays in OHSS, we determined the classic apoptosis related molecule levels in angiotensin II induced HUVECs treated with melatonin. The results demonstrated that incubation with angiotensin II (1 *μ*M) significantly increased the levels of p17 caspase 3 and decreased BCL2 level, which were partly restored by the treatment with melatonin (10 *μ*M)(*P* < 0.05) (Figures 4(a)4(b)). SESN2 has previous been demonstrated to be involved in the AMPK-mTOR signaling pathway which is related to apoptosis. Therefore, we studied this signal pathway and found that incubation with angiotensin II (1 *μ*M) in cultured HUVECs significantly increased the levels of p-AMPK and decreased the levels of p-mTOR and its downstream molecules (p-S6K1, p-4EBP1), which were partly restored by the treatment with melatonin (10 *μ*M) (*P* < 0.05) (Figures 4(a) and 4(b)). The same phenomenon was observed in KGNs (Figures 4(c) and 4(d)).

## 4. Discussion

In this study, we demonstrated that SESN2 level was increased in the granulosa cells of OHSS patients and was involved in the oxidative stress of OHSS by regulating the apoptosis of endothelium cells. Incubation with angiotensin II in cultured HUVECs and H_2_O_2_ in cultured KGNs induced augmented ROS generation and increased SESN2 expression, both of which were restored by the treatment of melatonin. These beneficial effects of melatonin could be explained partly by regulating antiapoptosis through SESN2-AMPK-mTOR.

OHSS is associated with more morbidity and mortality than other complications during IVF process. Despite strides to reduce the incidence of this potentially fatal and completely iatrogenic complication, it remains a serious health concern for a significant percentage of patients undergoing IVF. The prediction and prevention of OHSS are crucial for clinicians. In our research, OHSS patients exhibit significantly higher *E*_2_ levels on HCG day compared to non-OHSS patients, while basal *E*_2_ levels remain unchanged. FSH secreted by the pituitary gland stimulates the granulosa cells in antral follicles to produce and release *E*_2_ during the early follicular stage. *E*_2_, in turn, regulates the FSH level through negative feedback. In the early stages of decreased ovarian function, with a reduced number of antral follicles, the pituitary gland must secrete more FSH to maintain similar basal *E*_2_ levels. As a result, basal FSH levels increase while basal *E*_2_ levels remain the same. Most OHSS cases occur in women with better ovarian reserves, which explains why OHSS patients have lower basal FSH levels compared to non-OHSS women but higher AMH levels, while their basal *E*_2_ levels do not differ significantly, as demonstrated in our study. OHSS patients typically have better ovarian reserves and a greater number of ovarian follicles. During controlled ovarian stimulation with exogenous GnRH, their *E*_2_ levels on HCG day are higher than those of non-OHSS patients. It was previously thought that elevated plasma *E*_2_ concentrations or rapid increases in *E*_2_ levels during HCG ovulation induction indicated a higher sensitivity to HCG. However, it is now understood that *E*_2_ levels alone are not reliable predictors of OHSS [35, 36]. Our study corroborates previous research, demonstrating that it is not the basal *E*_2_ levels but rather the *E*_2_ levels on HCG day that can serve as predictors of OHSS [37, 38].

The development of OHSS is always accompanied by elevated *E*_2_ levels during the process of ART, and *E*_2_ has been implicated as a potential etiologic factor, one of the possible reasons is that elevated *E*_2_ increases capillary permeability so VEGF and other chemical mediators or precursors which augment fluid extravasation increase and thus OHSS developed. The overabundance of ROS released by VEGF and inflammatory factors causes the dilatation of endothelium, which leads to a massive shift of the body fluids from the vessels into the third space and increases the severity of inflammation and tissues injuries during the process of OHSS. Higher levels of melatonin are found in human follicular fluid than in plasma because melatonin is not only derived from the general circulation but also synthesized in the ovary [10]. Melatonin and its metabolites are free radical scavengers. Therefore, we speculate that the role of melatonin in the follicular fluid is to protect the oocytes and granulosa cells from being deteriorated by the oxidative stress and free radicals.

The stimulation and deterioration of vascular endothelial cells caused by ROS are the critical factors of OHSS. We found out that SESN2 level was closely associated with ROS, while melatonin could ameliorate SESN2 level induced by angiotensin II or H_2_O_2_. SESN2 is a cytoplasm stress-associated protein that accumulates in cells exposed to hypoxia, stress and DNA damage. Here, we demonstrated significantly higher level of SESN2 was expressed in high risk OHSS patients compared to that in controls, which may be attributed to an increased oxidative stress in OHSS patients. These results are constant with previous studies which showed that any condition which leads to ROS accumulation may induce SESN2 expression [19]. Oxidative stress drives endothelium deterioration, which contributes to the development and progression of OHSS. Anti-inflammatory and antioxidant agents prevent endothelium dilatation and reduce the oxidative imbalance in OHSS. Melatonin, as one of the most powerful free radical scavengers prevents granulosa cells in ovaries and endothelium from the deterioration of these free radicals.

The mechanisms by which melatonin provide antioxidant protection in OHSS are not fully understood. Previous studies have demonstrated that melatonin regulates antiapoptotic pathway [31–34]. BCL2 family and caspase family are important for regulating the atresia of antral follicles. Deletion of BCL2 increases the apoptosis of preantral follicle while the overexpression of caspases results in increased number of apoptotic follicles [10]. Our study demonstrated that the p17 caspase 3 level was increased while BCL2 level was decreased in OHSS oxidative models, which testified that the apoptosis signaling pathway was activated in OHSS oxidative models. Melatonin, could partly reverse p17 caspase 3 and BCL2 levels in OHSS oxidative model, which revealed the antioxidant effect of melatonin in OHSS by preventing oxidative stress-mediated apoptosis.

In recent years, more and more studies have shown that AMPK-mTOR molecules were also closely associated with apoptosis. Chen et al. have shown that H_2_O_2_ could induce apoptosis of neurons through activating AMPK and inhibiting mTOR [20]. Arsikin et al. have also demonstrated that 6-hydroxydopamine induced apoptosis through AMPK in SH-SY5Y neuroblastoma cells [39]. Our study demonstrated that ROS could active SESN2-AMPK-mTOR which resulted in the apoptosis in OHSS oxidative models. Melatonin prevents degenerative diseases in the nervous system by inhibiting apoptosis through regulation of the mTOR-mediated pathway.

The study has a few limitations. First, we selected KGNs as our human granulosa cell line to construct our OHSS oxidative models. However, it would be more accurate to validate our findings using other granulosa cell lines, such as SVOGs. Second, our results cannot be immediately applied to humans without the conduction of large-scale clinical trials. Further research is necessary to determine the precise dosage of melatonin in OHSS patients.

## 5. Conclusion

The results described herein help us understand the beneficial effects of melatonin in OHSS patients. The antioxidative and antiapoptotic properties of melatonin seem to produce positive effects on OHSS. Considering the safety of exogenous melatonin has been testified in many studies [40–42], our present findings will provide us the potential for clinical application of melatonin to prevent OHSS and define the most appropriate timing when the administration of melatonin should be effectively carried. This is the very initial step of our study and our results may not be adapted to OHSS patients without large-scale clinical studies. In our further study, we should focus on the exact mechanisms of the protective role of melatonin to support our study and large-scale clinical studied should be conducted to explore the long-term effects, way of administration, and appropriate drug dosage of melatonin in OHSS patients.

## Figures and Tables

**Table 1:** Clinical characteristics of the study population.

Parameters	Control (*n* = 15)	OHSS (*n* = 24)	*P* value
Age (years)	30.13 ± 3.54	27.08 ± 3.17	0.025^*∗*^
BMI (kg/m^2^)	22.78 ± 2.83	20.37 ± 2.00	0.017^*∗*^
AMH (ng/ml)	2.81 ± 1.07	13.34 ± 5.68	0.001^*∗∗*^
bFSH (mIU/ml)	7.06 ± 1.23	5.75 ± 1.06	0.006^*∗∗*^
bE_2_ (pg/ml)	40.71 ± 20.02	42.30 ± 18.02	0.828
E_2_ on hCG day (pg/ml)	2426.53 ± 1325.46	8589.23 ± 2623.04	0.001^*∗∗*^
Number of oocytes retrieved (n.)	10.87 ± 5.15	37.92 ± 11.00	0.001^*∗∗*^

^*∗*^ *P* < 0.05 compared with control group; ^*∗∗*^*P* < 0.01 compared with control group.

## Data Availability

All data used to support the findings of this study are available from the corresponding author upon reasonable request.

## References

[1] Mascarenhas M., Flaxman S., Boerma T., Vanderpoel S., Stevens G. A. (2012). National, regional, and global trends in infertility prevalence since 1990: a systematic analysis of 277 health surveys. *PLoS Medicine*.

[2] Asch R. H., Li H. P., Balmaceda J. P., Weckstein L. N., Stone S. C. (1991). Severe ovarian hyperstimulation syndrome in assisted reproductive technology: definition of high risk groups. *Human Reproduction*.

[3] Steward R. G., Lan L., Shah A. A. (2014). Oocyte number as a predictor for ovarian hyperstimulation syndrome and live birth: an analysis of 256381 in vitro fertilization cycles. *Fertility and Sterility*.

[4] Mocanu E., Redmond M. L., Hennelly B., Collins C., Harrison R. (2007). Odds of ovarian hyperstimulation syndrome (OHSS)-time for reassessment. *Human Fertility*.

[5] Rotshenker-Olshinka K., Badeghiesh A., Volodarsky-Perel A., Steiner N., Suarthana E., Dahan M. H. (2020). Trends in ovarian hyperstimulation syndrome hospitalization rates in the USA: an ongoing concern. *Reproductive BioMedicine Online*.

[6] Griesinger G., Diedrich K. (2009). Neue Entwicklungen bei der hormonellen Stimulation. *Gynäkologisch-Geburtshilfliche Rundschau*.

[7] Hortu I., Karadadas E., Ozceltik G. (2021). Oxytocin and cabergoline alleviate ovarian hyperstimulation syndrome (OHSS) by suppressing vascular endothelial growth factor (VEGF) in an experimental model. *Archives of Gynecology and Obstetrics*.

[8] Daghestani M. H., Alqahtani H. A., AlBakheet A. (2022). Global transcriptional profiling of granulosa cells from polycystic ovary syndrome patients: comparative analyses of patients with or without history of ovarian hyperstimulation syndrome reveals distinct biomarkers and pathways. *Journal of Clinical Medicine*.

[9] Gómez R., Soares S. R., Busso C., Garcia-Velasco J. A., Simón C., Pellicer A. (2010). Physiology and pathology of ovarian hyperstimulation syndrome. *Seminars in Reproductive Medicine*.

[10] Brzezinski A., Seibel M. M., Lynch H. J., Deng M. H., Wurtman R. J. (1987). Melatonin in human preovulatory follicular fluid. *Journal of Clinical Endocrinology and Metabolism*.

[11] Hattori A. (2007). The basic information for melatonin. *Modern Physician*.

[12] Zavodnik I. B., Domanski A. V., Lapshina E. A., Bryszewska M., Reiter R. J. (2006). Melatonin directly scavenges free radicals generated in red blood cells and a cell-free system: chemiluminescence measurements and theoretical calculations. *Life Sciences*.

[13] Lin T., Lee J. E., Kang J. W. (2009). Effects of melatonin on in vitro maturation of porcine oocyte and expression of melatonin receptor DNA in cumulus and granulosa cells. *Journal of Pineal Research*.

[14] Tamura H., Takasaki A., Miwa I. (2008). Oxidative stress impairs oocyte quality and melatonin protects oocytes from free radical damage and improves fertilization rate. *Journal of Pineal Research*.

[15] Velasco-Miguel S., Buckbinder L., Jean P. (1999). PA26, a novel target of the p53 tumor suppressor and member of the GADD family of DNA damage and growth arrest inducible genes. *Oncogene*.

[16] Querfurth W., LaFerla M. (2010). Alzheimer’s disease. *New England Journal of Medicine*.

[17] Peng M., Yin N., Li M. (2014). Sestrins function as guanine nucleotide dissociation inhibitors for Rag GTPases to control mTORC1 signaling. *Cell*.

[18] Schapira H., Tolosa E. (2010). Molecular and clinical prodrome of Parkinson disease: implications for treatment. *Nature Reviews Neurology*.

[19] Lee J. H., Budanov A. V., Park E. J. (2010). Sestrin as a feedback inhibitor of TOR that prevents age-related pathologies. *Science*.

[20] Wullschleger S., Loewith R., Hall N. (2006). TOR signaling in growth and metabolism. *Cell*.

[21] Budanov A. V., Sablina A. A., Feinstein E., Koonin E. V., Chumakov P. M. (2004). Regeneration of peroxiredoxins by p53-regulated sestrins, homologs of bacterial AhpD. *Science*.

[22] Kopnin P. B., Agapova L. S., Kopnin B. P., Chumakov P. M. (2007). Repression of sestrin family genes contributes to oncogenic ras-induced reactive oxygen species up-regulation and genetic instability. *Cancer Research*.

[23] Daixing Z., Chengye Z., Qiang Z., Shusheng L. (2013). Upregulation of sestrin-2 expression via P53 protects against 1-methyl-4-phenylpyridinium (MPP+) neurotoxicity. *Journal of Molecular Neuroscience*.

[24] Chen S., Yan W., Lang W. (2017). SESN2 correlates with advantageous prognosis in hepatocellular carcinoma. *Diagnostic Pathology*.

[25] Practice Committee of the American Society for Reproductive Medicine (2016). Prevention and treatment of moderate and severe ovarian hyperstimulation syndrome: a guideline. *Fertility and Sterility*.

[26] Gaytan F., Morales C., Roa J., Tena-Sempere M. (2018). Changes in keratin 8/18 expression in human granulosa cell lineage are associated to cell death/survival events: potential implications for the maintenance of the ovarian reserve. *Human Reproduction*.

[27] Wang N., Li H., Zhu Y., Li N., Chen Z. J., Zhang C. (2020). Melatonin protects against Epirubicin-induced ovarian damage. *Journal of Reproduction and Development*.

[28] Guo T., Zhang L., Cheng D. (2015). Low-density lipoprotein receptor affects the fertility of female mice. *Reproduction, Fertility and Development*.

[29] Cui L. L., Yang G., Pan J., Zhang C. (2011). Tumor necrosis factor *α* knockout increases fertility of mice. *Theriogenology*.

[30] Guo S., Yan X., Shi F., Ma K., Chen Z. J., Zhang C. (2018). Expression and distribution of the zinc finger protein, SNAI3, in mouse ovaries and pre-implantation embryos. *Journal of Reproduction and Development*.

[31] Ma W., He F., Ding F. (2018). Pre-treatment with melatonin enhances therapeutic efficacy of cardiac progenitor cells for myocardial infarction. *Cellular Physiology and Biochemistry*.

[32] Shi L., Liang F., Zheng J. (2018). Melatonin regulates apoptosis and autophagy via ROS-MST1 pathway in subarachnoid hemorrhage. *Frontiers in Molecular Neuroscience*.

[33] Segovia-Roldan M., Diez E., Pueyo E. (2021). Melatonin to rescue the aged heart: antiarrhythmic and antioxidant benefits. *Oxidative Medicine and Cellular Longevity*.

[34] Mannino G., Pernici C., Serio G., Gentile C., Bertea C. M. (2021). Melatonin and phytomelatonin: chemistry, biosynthesis, metabolism, distribution and bioactivity in plants and animals—an overview. *International Journal of Molecular Sciences*.

[35] Sood A., Goel A., Boda S., Mathur R. (2022). Prediction of significant OHSS by ovarian reserve and ovarian response-implications for elective freeze-all strategy. *Human Fertility*.

[36] Ocal P., Sahmay S., Cetin M., Irez T., Guralp O., Cepni I. (2011). Serum anti-Müllerian hormone and antral follicle count as predictive markers of OHSS in ART cycles. *Journal of Assisted Reproduction and Genetics*.

[37] Lee T. H., Liu H., Huang C. (2007). Serum anti-mullerian hormone and estradiol levels as predictors of ovarian hyperstimulation syndrome in assisted reproduction technology cycles. *Human Reproduction*.

[38] Aboulghar M. (2003). Prediction of ovarian hyperstimulation syndrome (OHSS). Estradiol level has an important role in the prediction of OHSS. *Human Reproduction*.

[39] Arsikin K., Kravic-Stevovic T., Jovanovic M. (2012). Autophagy-dependent and independent involvement of AMP-activated protein kinase in 6-hydroxydopamine toxicity to SH-SY5Y neuroblastoma cells. *Biochimica et Biophysica Acta-Molecular Basis of Disease*.

[40] Jan E., Wasdell B., Reiter J. (2007). Melatonin in therapy at pediatric sleep disorders: recent advances, why it works, who are the candidates and how to treat. *Current Pediatric Reviews*.

[41] Minich D. M., Henning M., Darley C., Fahoum M., Schuler C. B., Frame J. (2022). Is melatonin the “next vitamin D”?: a review of emerging science, clinical uses, safety, and dietary supplements. *Nutrients*.

[42] Guan Q., Wang Z., Cao J., Dong Y., Chen Y. (2021). Mechanisms of melatonin in obesity: a review. *International Journal of Molecular Sciences*.

[43] Zheng M., Liu M., Zhang C. (2002). Melatonin ameliorates ovarian hyperstimulation syndrome (OHSS) through SESN2 regulated antiapoptosis. https://europepmc.org/article/ppr/ppr481876.

